# A Naphthalimide-Based Fluorescence “Off-on-Off” Chemosensor for Relay Detection of Al^3+^ and ClO^−^

**DOI:** 10.3389/fchem.2019.00549

**Published:** 2019-08-02

**Authors:** Xue-Jiao Sun, Ting-Ting Liu, Hong Fu, Na-Na Li, Zhi-Yong Xing, Fan Yang

**Affiliations:** Department of Applied Chemistry, College of Science, Northeast Agricultural University, Harbin, China

**Keywords:** chemosensor, naphthalimides, Al^3+^, ClO^−^, logic gate

## Abstract

A novel Al^3+^ chemosensor **NPA** was designed and synthesized on basis of the mechanism of ICT and CHEF. Upon addition of Al^3+^, the probe **NPA** displayed a bright green fluorescence under UV radiation and visual color change from yellow to colorless. Spectrum titrations showed that **NPA** could be recognized as a fluorescent turn-on probe with 10^−8^ M detection level. The probe was successfully applied in real water sample and test paper. More important, **NPA**-Al^3+^ complex were used as a fluorescent turn-off probe for the detection of ClO^−^ with the detection as low as 2.34 × 10^−8^ M. The performance of **NPA** to Al^3+^ and **NPA**-Al^3+^ complex to ClO^−^ demonstrated that **NPA** could be served as a sensitive probe and exhibit INHIBIT logic gate behavior with Al^3+^ and ClO^−^ as inputs.

## Introduction

Aluminum, the third most abundant metal element in the Earth's crust, is widely used in human's daily life including pharmaceuticals, textile, kitchen utensils, and paper industries (Zhang et al., 2016; Kaur and Kaur, 2017). Moreover, Al^3+^ ion widely exists in the environment, normally in natural waters and many plants, which can enter the human body through foods and water. However, excess aluminum can damage the human nervous system, and has tightly relation to many diseases such as Alzheimer's disease, Parkinson's disease, anemia, dementia, encephalopathy, and gastrointestinal diseases (Helal et al., 2013; Liu et al., 2014, 2017; Jiang et al., 2015; Zeng et al., 2018). In addition, hypochlorous acid (HOCl), known as an important reactive oxygen species (ROS) in many living organisms, plays a vital role in many biological processes (Zhu et al., 2014). Hypochlorite is a key microbicide that is used for natural defense because it behaves as a strong nucleophilic non-radical oxidant (Shi et al., 2010). However, it also results in many pathological diseases, especially is implicated in inflammation-associated injury including hepatic ischemia-reperfusion injury, lung injury, rheumatoid arthritis, and atherosclerosis. Moreover, excessive or misplaced of hypochlorite can also cause detrimental effect on tissues (Lei et al., 2017; Lin et al., 2017; Shen et al., 2017). Therefore, the determination of Al^3+^ and ClO^−^ in biological samples is of great importance.

In the last few decades, the designing a molecular system, which displayed significant changes in electronic, magnetic, or optical signals even at low concentration during the detection for a specific guest species, was a hot topic for many researchers (Kim et al., 2013; Liu et al., 2014; Kang Y. et al., 2016; Lim et al., 2017). Among them, small-molecule optical probes, which exhibited many merits including high selectivity and sensitivity, tunability, simple manipulation, and direct visualization, were employed as a powerful tool in the facet of trace analysis and rapid detection for various analytes (Qin and Yang, 2015; Wang et al., 2015; Simon et al., 2016; Liu et al., 2017; Murugan et al., 2017). Up to now, lots of papers that one probe only for the detection of one special analyte such as Al^3+^ ion (Tang et al., 2015; Gupta and Kumar, 2016; Xie et al., 2017) or Hypochlorous acid (Zhang et al., 2017) had been reported, but the design idea, one fluorescent probe for successively recognition of two different analytes, had gained increasing attention in considering its high efficiency and potential cost reduction (Wang et al., 2014; Ye et al., 2014; Zhao et al., 2015; Wen and Fan, 2016; Xie et al., 2016; Zhai et al., 2016; Zhu et al., 2016; Wu et al., 2017). Many excellent probes had been reported for relay recognition of two different ions through fluorescent “off-on-off” (Borasea et al., 2015; He et al., 2015; Zhao et al., 2015; Rai et al., 2016; Bhattacharyya et al., 2017; Das et al., 2017; Jo et al., 2017; Dwivedi et al., 2018; Feng et al., 2018; Lim et al., 2018) or “on-off-on” (Diao et al., 2016; Zhao et al., 2016; Sarkar et al., 2017) mode. Moreover, 1, 8-naphthalimide fluorophore, holding many excellent photophysics properties, such as high photostability, visible absorption and fluorescence emission, large Stokes' shift and high fluorescence quantum yield (Dimov et al., 2014; Yu and Zhang, 2014; Kang L. et al., 2016; Li et al., 2018; Fu et al., 2019), was successfully applied in designing fluorescent probes toward various analytes.

Taking above statements into consideration, in this paper, we prepared and characterized a 1, 8-naphthalimide-based fluorescent probe **NPA** for the detection of Al^3+^ embodied in colorimetric and fluorescent turn-on was ascribed to the co-contribution of intramolecular charge transfer (ICT) and chelation enhanced fluorescence (CHEF). The binding stoichiometry between the **NPA** and Al^3+^ had been clarified according to various spectroscopic measurements and data analysis. Moreover, the performance of *in situ* formed **NPA**-Al^3+^ complex for the detection of ClO^−^ was investigated. The **NPA**-Al^3+^ complex exhibited a fluorescent turn-off response in the detection of ClO^−^. Inspiringly, to the best of our knowledge, a single probe for sequential detection of Al^3+^ and ClO^−^ through fluorescence “off-on-off” mode is scarcely documented.

## Materials and Methods

### Reagents and Instrument

All the solvents and reagents (analytical or spectroscopic grade) were purchased commercially and used as received. Metal salts [NaClO_4_, KClO_4_, Mg(ClO_4_)_2_, Ba(ClO_4_)_2_, Zn(ClO_4_)_2_•6H_2_O, Cu(ClO_4_)_2_•6H_2_O, AgNO_3_, Cd(NO_3_)_2_, Pb(NO_3_)_2_, Co(NO_3_)_2_•6H_2_O, Ni(NO_3_)_2_•6H_2_O, Ca(NO_3_)_2_•4H_2_O, Al(NO_3_)_3_•9H_2_O, MnSO_4_•H_2_O, HgCl_2_, FeCl_2_•4H_2_O] were obtained from commercial suppliers, and used as received without further purification. ^1^H NMR and ^13^C NMR spectra were recorded on a AV- 600 spectrometer in DMSO-d_6_ solution. The chemical shifts (δ) are reported in ppm and coupling constants (*J*) in Hz relative to TMS (0.00) for ^1^H NMR and ^13^C NMR. Mass spectra were measured on a Waters Xevo UPLC/G2-SQ Tof MS spectrometer. Absorption spectra were recorded using a Pgeneral TU-1901 UV-vis spectrophotometer. Fluorescence measurements were performed on a Perkin Elmer LS55 fluorescence spectrometer at room temperature.

### Synthesis of the Probe NPA

**Scheme 1:**
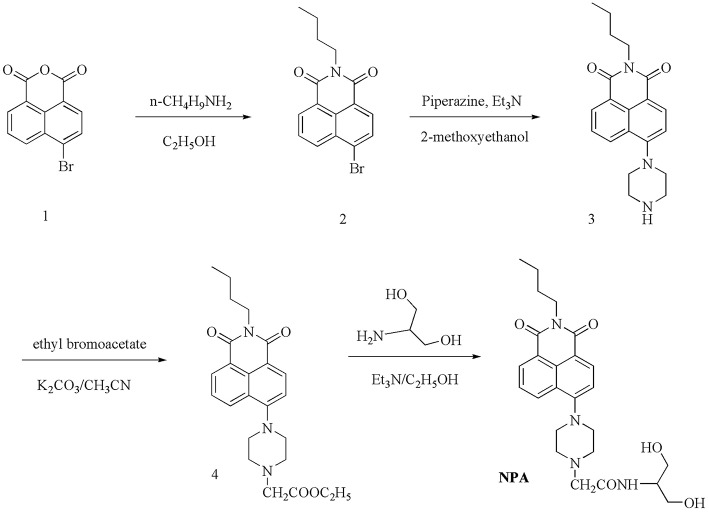
Synthesis of probe **NPA**.

**NPA** was prepared in four steps from naphthalimide as the starting material, as shown in Scheme 1. Compound 1–4 were synthesized using the literature method (Kang L. et al., 2016; Li et al., 2018).

The compound 4 (330 mg, 0.97 mmol) and 2-amino-propane-1, 3-diol (72 mg, 0.8 mmol) were dissolved in ethanol (45 mL) and stirred over anhydrous Et_3_N (80 mg, 0.58 mmol) for 24 h at room temperature. The crude product was purified by column chromatography with CH_3_OH/CHCl_3_ (1:20, v/v) as the eluent and further purified by recrystallization from ethanol to get orange-yellow solid **NPA** (210 mg, yield: 46%); m.p: 195–196°C.^1^HNMR (600 MHz, DMSO) (Supplementary Figure 1): δ (ppm) (d, J = 7.2 Hz, 1H), 8.43 (d, J = 8.5 Hz, 1H), 8.39 (d, J = 8.1 Hz, 1H), 7.83–7.75 (m, 1H), 7.50 (d, J = 8.5 Hz, 1H), 7.35 (d, J = 8.1 Hz, 1H), 4.71 (t, J = 5.4 Hz, 2H), 4.07–3.97 (m, 2H), 3.82–3.72 (m, 1H), 3.54–3.45 (m, 2H), 3.46–3.38 (m, 2H), 3.27 (s, 4H), 3.10 (s, 2H), 2.80 (s, 4H), 1.64–1.54 (m, 2H), 1.39–1.30 (m, 2H), 0.92 (t, J = 7.4 Hz, 3H). ^13^C NMR (151 MHz, DMSO) (Supplementary Figure 2): δ (ppm) 168.62, 163.32, 162.81, 155.32, 131.96, 130.42, 130.26, 128.89, 125.84, 125.10, 122.37, 115.42, 114.92, 60.86, 59.69, 52.53, 52.40, 51.91, 38.91, 29.51, 19.59, 13.52. MS (ESI) (Supplementary Figure 3): m/z [**NPA**+H^+^]^+^ calcd 469.2451, found 469.2458.

### Preparation of Solutions for Spectral Detection

All stock solutions (10 mM) including metal cations and the anions were prepared with distilled water, while the stock solution of compound **NPA** (0.1 mM) was prepared in CH_3_OH (100 mL). One milliliter of **NPA** solution (0.1 mM) was diluted in 9 mL CH_3_OH to make the test solutions (10 μM). For fluorescence measurements, excitation was set at 400 nm, and the excitation and emission slit widths were 10 and 10 nm, respectively. All spectroscopic measurements were performed in CH_3_OH at room temperature.

Stock solution of **NPA** (0.1 mM) was prepared by **NPA** (0.01 mmoL) was dissolved in CH_3_OH (100 mL), the test solutions of **NPA** (10 μM) was prepared by adding 1 mL of **NPA** stock solution (0.1 mM) was diluted in 9 mL CH_3_OH in CH_3_OH.

Stock solutions (10 mM) including the metal cations and the anions were prepared with ultrapure water, respectively. For spectrum measurement, the test solutions were prepared by adding certain amount of stock solution using a pipette into **NPA** stock solution. For fluorescence measurements, excitation was set at 400 nm, and the excitation and emission slit widths were 10 and 10 nm, respectively.

### Determination of Binding Constant and Detection Limit

According to the fluorescence intensity data, the binding constant of **NPA** with Al^3+^ was calculated based on the modified Benesi–Hildebrand equation (Li et al., 2018) as followed. Where, *F*_max_, *F* and *F*_min_ are the fluorescence intensities of **NPA** in the presence of Al^3+^ at saturation, at an intermediate Al^3+^ concentration, and absence of Al^3+^, respectively. *K* is the stability constant.

$$\begin{array}{l} \frac{1}{F-F_{min}}=\frac{1}{K\left(F_{max}-F_{min}\right)\left[Al^{3+}\right]}-\frac{1}{F_{max}-F_{min}} \end{array}$$

The limit of detection (LOD) of Al^3+^ was calculated on the basis of 3δ/K according to the fluorescence changes, δ is the standard deviation of the blank solution, and K is slope of calibration curve (Borasea et al., 2015; Zeng et al., 2018).

## Results and Discussion

### The UV-Vis Spectra Responses of Probe NPA

**Figure 1:**
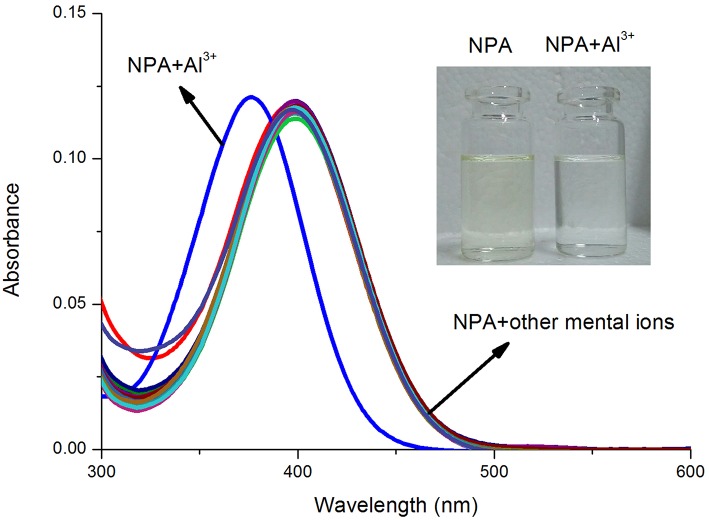
UV-vis absorbance spectra of **NPA** (10 μM) in the absence and presence of various mental ions (50 μM) in CH_3_OH. Inset: The color change of **NPA** (10 μM) in the presence of Al^3+^ ions (5 equiv.) in CH_3_OH under natural light.

Various metal ions: K^+^, Na^+^, Ca^2+^, Mg^2+^, Ba^2+^, Pb^2+^, Cu^2+^, Co^2+^, Ni^2+^, Fe^2+^, Cd^2+^, Hg^2+^, Mn^2+^, Ag^+^, Zn^2+^, and Al^3+^ were used to observe the selectivity of probe **NPA** (10 μM) and their UV-vis spectra were measured in CH_3_OH. As shown in Figure 1, **NPA** exhibited a characteristic absorbance band at 400 nm in the presence of tested metal ions (K^+^, Na^+^, Ag^+^, Ca^2+^, Mg^2+^, Ba^2+^, Pb^2+^, Cu^2+^, Co^2+^, Ni^2+^, Fe^2+^, Cd^2+^, Hg^2+^, Mn^2+^, Zn^2+^) and there was almost no difference compared with that of **NPA** itself. However, upon the addition of Al^3+^, the maximum absorbance peak of **NPA** was blue-shifted with 21 nm from 399 to 378 nm, and the solution color was changed from yellow to colorless. This result might attribute to the decrease of conjugated degree resulting from the decrease of electron donating ability of piperazine ring to the naphthalimide after the complexation with Al^3+^ (De Silva et al., 1997; Urano et al., 2015; Kang L. et al., 2016).

**Figure 2:**
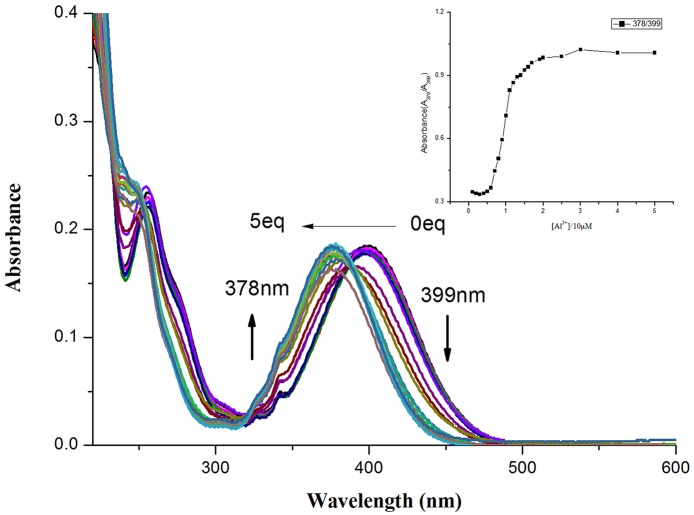
UV-vis spectral of **NPA** (10 μM) upon addition of increasing concentration of Al^3+^ (0–5 equiv.) in CH_3_OH. Insert: Plot of absorbance intensity ratio (A_378_/A_399_) as a function of the Al^3+^ concentration.

In addition, the quantitative sensing of Al^3+^ ion was elucidated by UV-vis titration of probe **NPA** in CH_3_OH solution (Figure 2). **NPA** alone showed a major absorbance band at 399 nm, a new band at 378 nm appeared upon addition of Al^3+^ and the intensity increased gradually and then kept constant until the amount added of Al^3+^ was more than 20 μM. The good relationship was found between the ratio of absorbance (A_378_/A_399_) vs. the concentration of Al^3+^ (Supplementary Figure 4), and the limit of detection (LOD) for Al^3+^ was calculated as 3.61 × 10^−8^ M on the basis of 3 δ/K (where δ is deviation of the blank signal and K is slope of calibration curve; Borasea et al., 2015; Zeng et al., 2018). These results indicated that **NPA** could be used as an absorbance-ratiometric probe for the detection of Al^3+^.

### Fluorescence Spectral Responses of NPA

**Figure 3:**
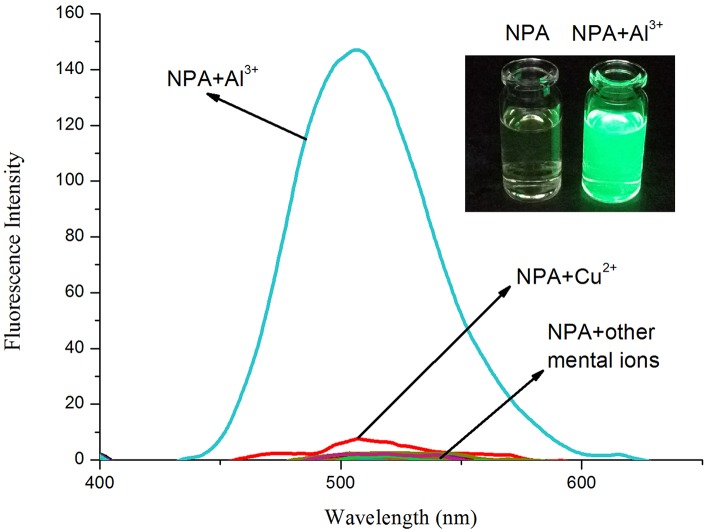
Fluorescence spectral changes (λ_ex_ = 400 nm) of **NPA** (10 μM) in the presence of various mental ions (50 μM) in CH_3_OH. Inset: The color change of **NPA** (10 μM) in the presence of Al^3+^ ions (5 equiv.) in CH_3_OH under 365 nm UV lamp.

The fluorescence properties of **NPA** were investigated in the presence of a variety of metal ions in CH_3_OH (Figure 3). **NPA** itself exhibited almost no fluorescence emission upon excitation at 400 nm. However, the addition of Al^3+^ (5 equiv.) into **NPA** induced an obvious fluorescence enhancement at 505 nm, which might be attributed to the CHEF effect through the formation of a rigid system after binding with Al^3+^ (Kang L. et al., 2016; Zeng et al., 2019). In contrast, few fluorescence changes were observed in the presence of other tested metal ions, demonstrating that **NPA** was highly selective for Al^3+^ over competing metal ions.

**Figure 4:**
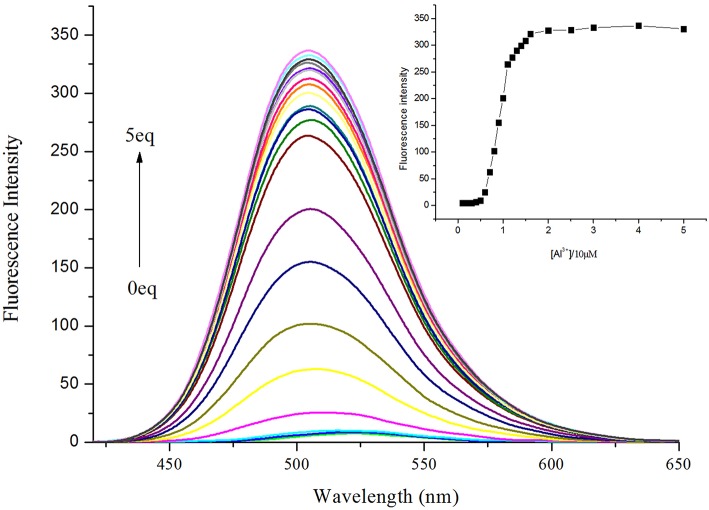
Fluorescence emission spectral (λ_ex_ = 400 nm) of **NPA** (10 μM) with increasing concentration of Al^3+^ in CH_3_OH. Inset: Plot of fluorescence intensity at 505 nm as a function of the Al^3+^ concentration.

To further validate the utility of probe **NPA**, the fluorescence titrations of **NPA** were performed by gradually increasing the concentration of Al^3+^ (Figure 4). The emission intensity of **NPA** at 505 nm progressively increased upon addition of Al^3+^, and the emission intensity remained constant when the quantity of Al^3+^ added was over 15 μM (Figure 4, Insert). Moreover, the fluorescence enhancement of sensor **NPA** depending on the concentration of Al^3+^ was in a linear manner (Supplementary Figure 5). The detection limit (LOD) for Al^3+^ ion according to fluorescence changes was measured to be 2.03 × 10^−8^ M on the basis of 3 δ/K (Borasea et al., 2015; Zeng et al., 2018). The result clearly demonstrated that the probe **NPA** was highly efficient in sensing Al^3+^ at nanomolar level.

**Figure 5:**
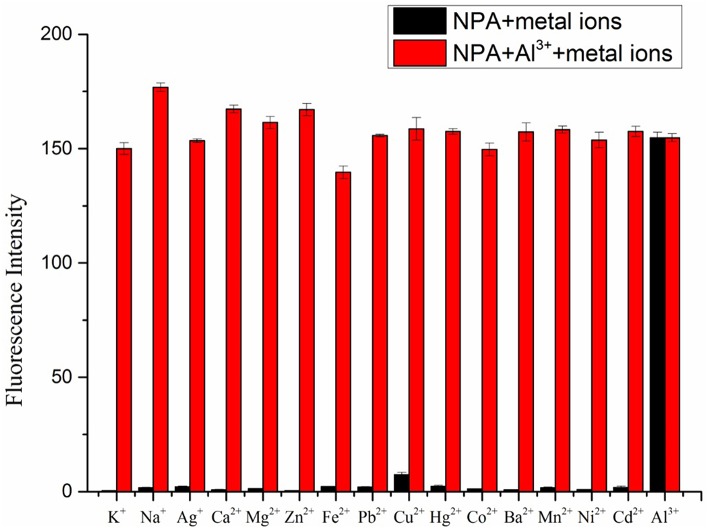
Fluorescence intensity of **NPA** (10 μM) at 505 nm upon addition of Al^3+^ (50 μM) in the presence of various mental ions (50 μM) (λ_ex_ = 400 nm). (Error bar was represented as mean ± standard deviation, *n* = 3).

Further, tolerance of fluorescence intensity of **NPA**-Al^3+^ complex in presence of other metal ions was tested (Figure 5). All competitive metal ions had no obvious interference on the Al^3+^ detection, indicated that **NPA**-Al^3+^ complex was hardly affected by these coexistent metal ions. Accordingly, **NPA** can be used as selective fluorescent probe for Al^3+^ determination without disturbance of other competing metal ions.

### Response Time Studies

As for an excellent fluorescent probe, fluorescent stability and response time are two crucial factors. Hence, the changes of fluorescent intensity of **NPA** and the response time of **NPA** to Al^3+^ were investigated (Supplementary Figure 6). The results demonstrated that the fluorescence signal of **NPA** remained stable for a long time in the absence of Al^3+^, implying the **NPA** had good fluorescence stability. Moreover, after addition of Al^3+^, the fluorescence intensity of **NPA** at the 505 nm reached the maximum within 10 s and maintained constant more than 3 min, indicating the unique feature of high complexation ability of **NPA** with Al^3+^.

### Binding Stoichiometry and Sensing Mechanism

**Figure 6:**
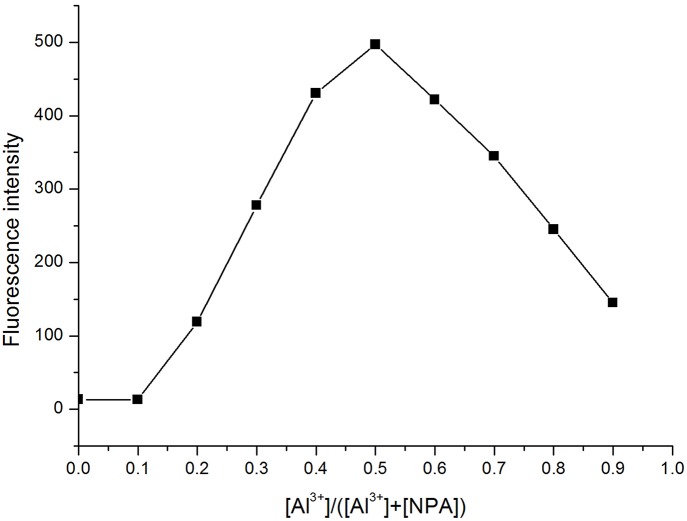
Job's plot of **NPA** with Al^3+^ in CH_3_OH. {[Al^3+^]/([Al^3+^] + [**NPA**])} is the molar fraction of Al^3+^ ion.

**Figure 7:**
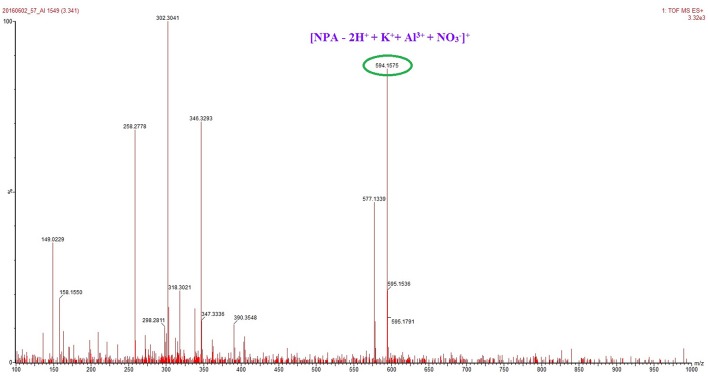
ESI–MS spectrum of **NPA** (50 μM) upon addition of 5 equiv. of Al^3+^ in CH_3_OH.

The total concentration of Al^3+^ and ligand was 50 μM with the molar ratio of Al^3+^ changed from 0.1 to 0.9. The fluorescence mission was measured for each sample in CH_3_OH with the excitation wavelength at 400 nm. The maximum point appeared at a mole fraction of 0.5 (Figure 6), indicating a 1:1 stoichiometry of the binding mode between **NPA** and Al^3+^, and which was further clarified by a peak at *m*/*z* 594.1575, which was assignable to **[NPA** – 2H^+^ + K^+^+ Al^3+^ + ${\text{NO}}_{3}^{-}$**]**^**+**^ (calcd. *m*/*z* 594.1547) in the ESI mass spectrum (Figure 7).

According to the above results, the association constant was calculated as 7.06 × 10^4^ M^−1^ according to the fluorescence titration data (Supplementary Figure 7), basing on the Benesi-Hildebrand plot (Li et al., 2018).

**Figure 8:**
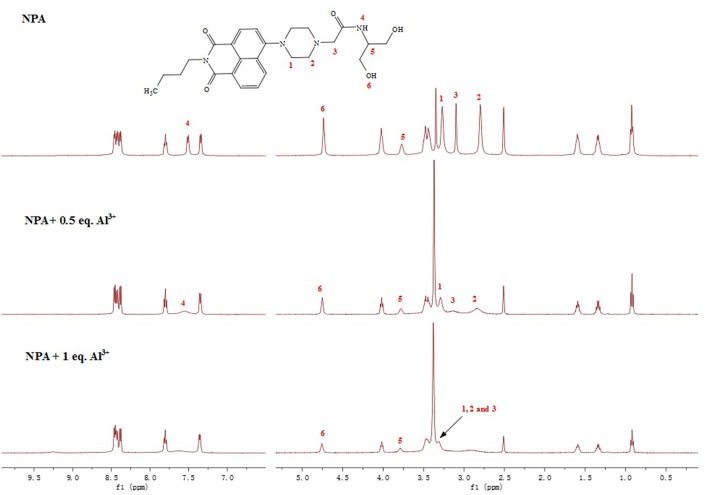
^1^H NMR spectra of **NPA** with Al^3+^ in DMSO *d*_6_.

**Scheme 2:**
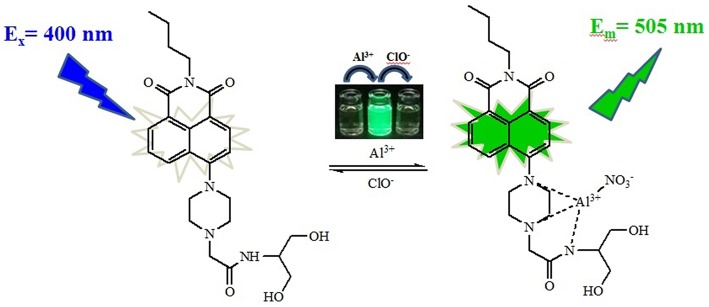
Proposed mechanism for the fluorescent sensing of **NPA** and its *in situ* complex with Al^3+^ and ClO^−^.

To better evaluate the interaction of **NPA** with Al^3+^, ^1^H NMR spectra of **NPA** were constructed in the absence and presence of different equivalent Al^3+^ in DMSO (Figure 8). The protons of H_1_ and H_2_ of **NPA** at around 2.80 and 3.27 ppm were shifted downfield to 3.42 upon the addition of Al^3+^. In addition, the peak of **NPA** at around 3.10 ppm was also shifted downfield to 3.42 ppm, indicated that the two nitrogen atoms of piperazine ring might coordinate the Al^3+^. Moreover, the proton of amide was disappeared supporting the occurrence of deprotonation upon the interaction of amide with the Al^3+^. According to the above results, the two nitrogen atoms of piperazine ring and the amide nitrogen atom might coordinate with Al^3+^ (Scheme 2).

### Reversibility of NPA Toward Al^3+^

The fluorescence intensity enhancement of **NPA** on interaction with Al^3+^ was found to be reversible by using EDTA as the recovering reagent. When the strong metal ion chelating agent EDTA was added gradually to a mixture of **NPA**-Al^3+^, the UV-vis absorbance spectral and fluorescence spectral of the solution of **NPA**-Al^3+^ almost recovered to the original condition in the absence of Al^3+^ (Supplementary Figures 8, 9). These results indicating that recognition process can be made reversible merely by treatment with EDTA. Besides, as for an excellent chemosensor, reversibility and regeneration are crucial for the fabrication of apparatus to sense Al^3+^.

### Application of NPA for Al^3+^ Analysis in Test Paper

Interestingly, the noticeable colorimetric changes of the system and qualitative recognition of Al^3+^ in solution were confirmed by the simple test strips. The required test strips were immersed in CH_3_OH (10 mL) including Al^3+^ and then dried in air. The color changes in various concentrations of Al^3+^ under sunlight and under 365 nm UV light were illustrated in Supplementary Figure 10. Visual color changed from yellow to colorless and bright-green fluorescence increased gradually with increasing amounts of Al^3+^ were clearly observed. This result showed that **NPA** might be used as a portable detector for Al^3+^.

### Application of NPA for Al^3+^ Analysis in Water Samples

In order to verify the practical application of **NPA**, ultrapure water and tap water samples were analyzed by the proposed fluorimetric method. The results were summarized in Supplementary Table 1 with satisfactory recovery of Al^3+^, indicating that the present fluorescent probe seem to be applicable for the determination of Al^3+^ in environmental analysis.

### Fluorescent “On-Off” Sensing of ClO^−^ by NPA-Al^3+^ Complex

**Figure 9:**
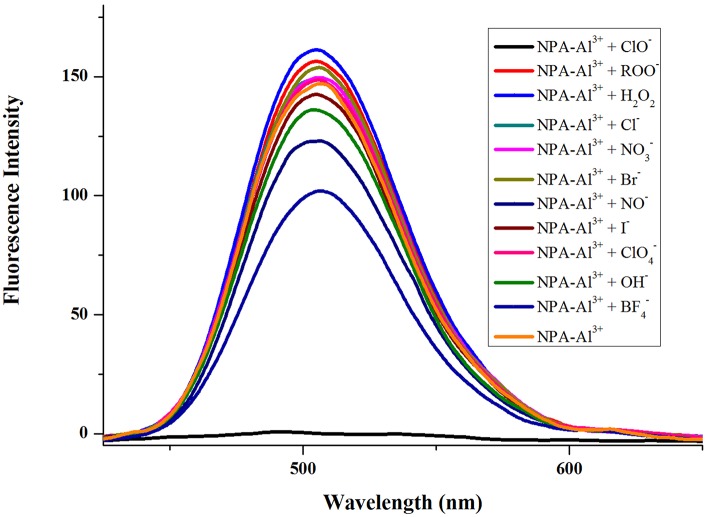
Fluorescence spectra (λ_ex_ = 400 nm) of **NPA**-Al^3+^ system in presence of various anions (100 μM) in CH_3_OH.

In order to further explore the performance of **NPA**-Al^3+^ complex to different anionic, the fluorescence spectra of **NPA**-Al^3+^ complex were investigated in presence of various 100 μM anions (ClO^−^, ROO^−^, H_2_O_2_, OH^−^, NO^−^, Cl^−^, Br^−^, ${\text{NO}}_{3}^{-}$, ${\text{BF}}_{4}^{-}$, ${\text{ClO}}_{4}^{-}$) in CH_3_OH (Figure 9). The result showed that fluorescence intensity of **NPA**-Al^3+^ complex was affected to tolerable degree by I^−^, H_2_O_2_, and ${\text{BF}}_{4}^{-}$ except for ClO^−^, which completely quenched the fluorescence of **NPA**-Al^3+^ complex. This result suggested that the **NPA**-Al^3+^ complex could distinguish ClO^−^ anion over other ROO^−^, H_2_O_2_, OH^−^, NO^−^, Cl^−^, Br^−^, ${\text{NO}}_{3}^{-}$, ${\text{BF}}_{4}^{-}$, ${\text{ClO}}_{4}^{-}$ by fluorescence “on-off” mode.

**Figure 10:**
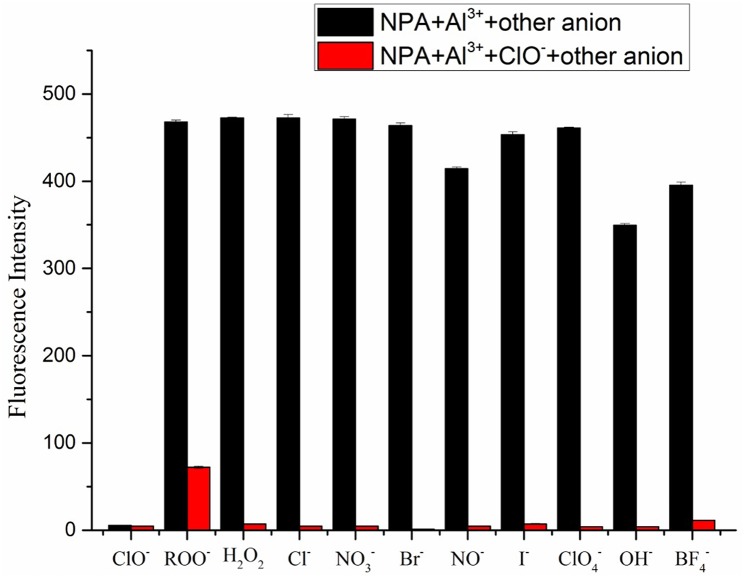
Changes in emission intensity of the **NPA**-Al^3+^ complex at 505 nm upon addition of 100 μM of various anions followed by addition of ClO^−^ (λ_ex_ = 400 nm). (Error bar was represented as mean ± standard deviation, *n* = 3).

Furthermore, competition experiments were carried out by addition of various anions (100 μM) to the solution of **NPA**-Al^3+^ in the presence of 100 μM of ClO^−^. As shown in Figure 10, competitive anion had no prominent interference with the determination of ClO^−^, which meant that **NPA**-Al^3+^ could perform as a highly selective probe for ClO^−^ via a fluorescence “turn-off” mechanism.

**Figure 11:**
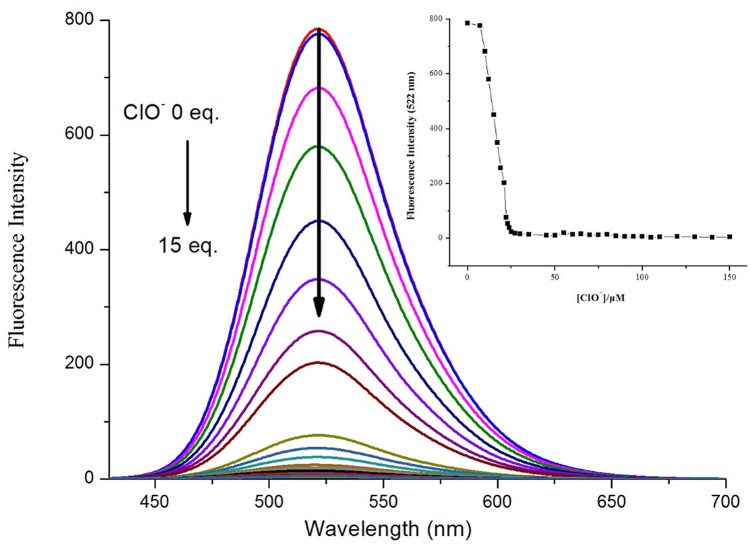
Fluorescence spectral (λ_ex_ = 400 nm) of **NPA**-Al^3+^ complex at different added concentration of ClO^−^ in CH_3_OH. Insert: Plot of fluorescence intensity of verse ClO^−^ concentration in in CH_3_OH.

**Figure 12:**
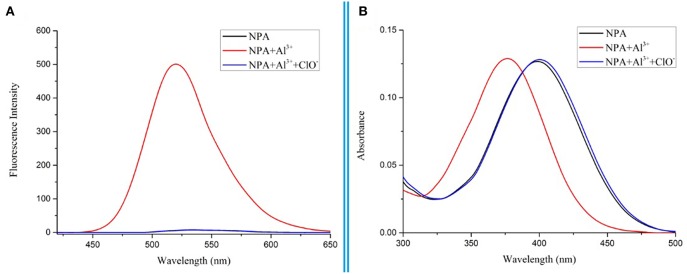
Fluorescence spectrum **(A)** and UV-vis absorbance spectrum **(B)** of **NPA** in CH_3_OH while adding Al^3+^ (3 eq.) and ClO^−^ (10 eq.) to the solution.

To further investigate the binding ability and limit of detection of the **NPA**-Al^3+^ complex with ClO^−^. The complex sensing capability was studied in detail analysis with fluorescence titration (Figure 11). Upon addition of ClO^−^ to the solution containing **NPA**-Al^3+^ complex, fluorescent intensity at 505 nm gradually diminishes, and 40 μM of ClO^−^ could lead to complete fluorescent quenching (Figure 11, inset). Obviously, the fluorescence intensity of **NPA**-Al^3+^ complex depending on the concentration of ClO^−^ was in a linear manner (Supplementary Figure 11). According to the fluorescence titration data, the detection limit of **NPA**-Al^3+^ complex was determined and be found to 2.34 × 10^−8^ M (Supplementary Table 3). Furthermore, fluorescence quenching *in situ* also indicated that the occurrence of dissociation of **NPA**-Al^3+^ complex, and the **NPA** was regenerated upon the addition of ClO^−^, which further supported by the verified experiments as followed. Firstly, the peak at *m*/*z* 469.2438 appeared in ESI–MS spectrum of **NPA-Al**^**3+**^ complex upon addition of ClO^−^ in CH_3_OH, which was assignable to [**NPA**+H^+^]^+^ (calcd *m*/*z* 469.2451; Supplementary Figure 12) compared with the ESI–MS spectrum of **NPA** itself (Supplementary Figure 13). Moreover, the UV-vis absorbance and fluorescence spectrum of **NPA**-Al^3+^ complex in the absence and presence of ClO^−^ were measured (Figure 12), respectively. The result showed that both of them were almost the same as that of **NPA** itself. Lastly, the titration of ^1^H NMR (Supplementary Figure 14) and ^13^C NMR (Supplementary Figure 15) were also investigated to clarify the sensing mechanism. The result displayed that **NPA** was regenerated to some extent upon the addition of ClO^−^ to the **NPA-Al**^**3+**^ complex. The above result implies that the **NPA**-Al^3+^ complex could act as a fluorescent turn-off probe for ClO^−^ recognition. According to above results, the “off-on-off” mechanism was achieved with sequence specificity (Al^3+^ and ClO^−^) in CH_3_OH (Scheme 2). In addition, the results of the comparison between **NPA** and those reported sensors (one fluorescent probe for successively recognition of two different analytes) were summarized in Supplementary Table 2.

### Application as Logic Gate Function

**Figure 13:**
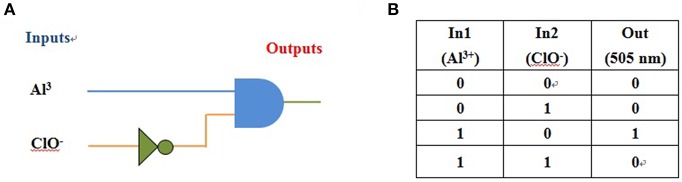
**(A)** The logic circuit displaying memory unit with two inputs (Al^3+^ and ClO^−^) and one output; **(B)** corresponding truth table.

**NPA** alone displayed very weak fluorescence emission. Maximum emission at 505 nm appeared after coordination of **NPA** with the Al^3+^. Moreover, when ClO^−^ was added to the above solution, the emission intensity at 505 nm was quenched. In addition, the fluorescence “off-on-off” response for Al^3+^ and ClO^−^ were carried out (Supplementary Figure 16), and the result showed that cycle times was more than 5 according to **NPA** fluorescent signal upon the alternate addition of Al^3+^ and ClO^−^. Due to the remarkable fluorescence changes of probe **NPA** in the presence Al^3+^ and ClO^−^, it would be able to construct a two output combinatorial logic circuit with two signal inputs that are input 1 (Al^3+^) and input 2 (ClO^−^; Figure 13A). Input 1 caused fluorescence enhancement, equivalent to a YES operation, while input 2 resulted in fluorescence quenching, thereby enforcement of the NOT gate. In the presence of both inputs, the quenching (by Input 2) has precedence over the fluorescence enhancement by Input 1, which was in accordance with the truth table illustrated in Figure 13B. Hence, by monitoring the emission maxima at 505 nm by two inputs (Al^3+^ and ClO^−^), a monomolecular circuit displaying an INHIBIT logic function could be achieved.

## Conclusion

In conclusion, we had developed a naphthalimide-based sensor **NPA** which exhibited a turn-on fluorescence response toward Al^3+^ with a bright green fluorescence under UV radiation and visual color change from yellow to colorless detected by naked-eye. The binding stoichiometry of **NPA** with Al^3+^ was 1:1 confirmed by job's plot, HRMS and ^1^H NMR and ^13^C NMR titration. The application of **NPA** for detection and assessing the existence of Al^3+^ in real sample was also successfully achieved. Moreover, **NPA**-Al^3+^ complex were further used as a fluorescent turn-off probe for the detection of ClO^−^ with the detection as low as 2.34 × 10^−8^ M. The INHIBIT molecular logic gate was effectively constructed by the performance of **NPA** to Al^3+^ and **NPA**-Al^3+^ complex to ClO^−^. The development of multi-functional chemosensor for the detection of biological-related analyst in pure water is our future work.

## Data Availability

The raw data supporting the conclusions of this manuscript will be made available by the authors, without undue reservation, to any qualified researcher.

## Author Contributions

Z-YX constructed the workflow and completed the paper. N-NL synthesized and purified the compound. X-JS and T-TL measured and interpreted the data the UV/Vis and fluorescence experiments. HF and FY performed the real sample analysis.

### Conflict of Interest Statement

The authors declare that the research was conducted in the absence of any commercial or financial relationships that could be construed as a potential conflict of interest.
