# Supplementary material for: A Naphthalimide-Based Fluorescence “Off-on-Off” Chemosensor for Relay Detection of Al3+ and ClO−
Source: Front Chem. 2019 Aug 2;7:549. doi: 10.3389/fchem.2019.00549 (PMC6687763; doi:10.3389/fchem.2019.00549)
Supplement: Supplementary file 1 [file Table_1.DOCX]

Supplementary Material

# Supplementary Figures and Tables

## Supplementary Figures


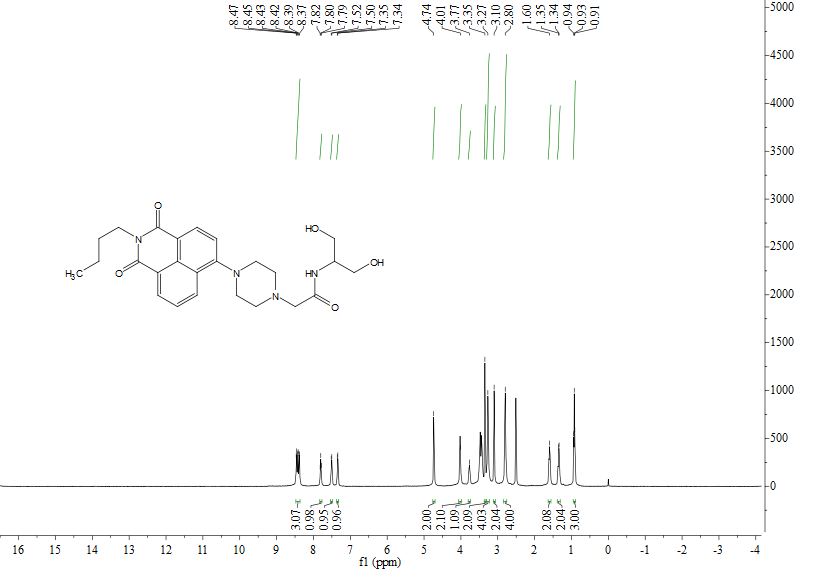


**Supplementary Figure 1.** ^1^H NMR spectra of **NPA** in DMSO.

**Supplementary Figure 2.**^13^C NMR spectra of **NPA** in DMSO.

**Supplementary Figure 3.** ESI-MS spectra of **NPA** in CH_3_OH.


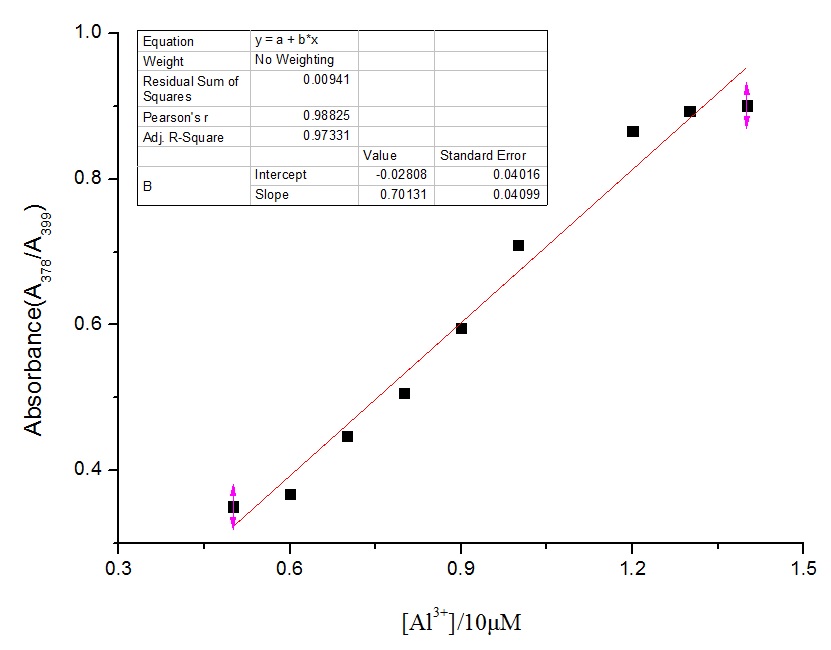


**Supplementary Figure 4.** Absorbance intensity at 378 nm/399 nm as a function of Al^3+^ (0.6–1.3 equiv.) in CH_3_OH


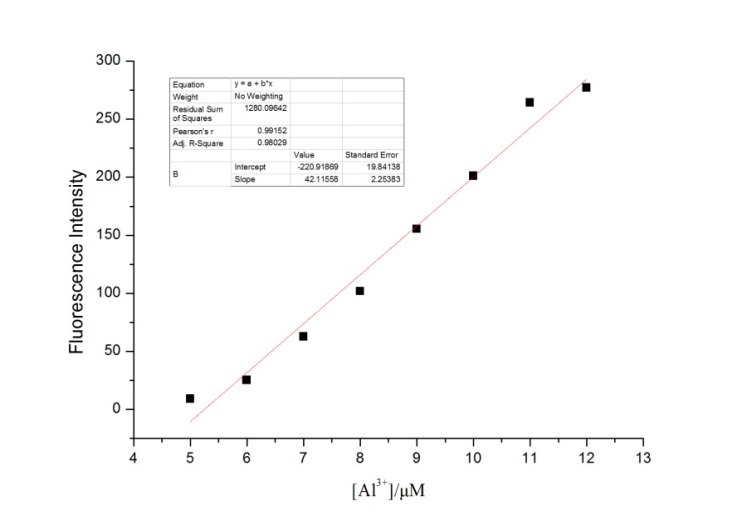


**Supplementary Figure 5.** Fluorescence intensity of **NPA** (10 μM) in presence of Al^3+^ (0.5 – 1.2 equiv.) in CH_3_OH.


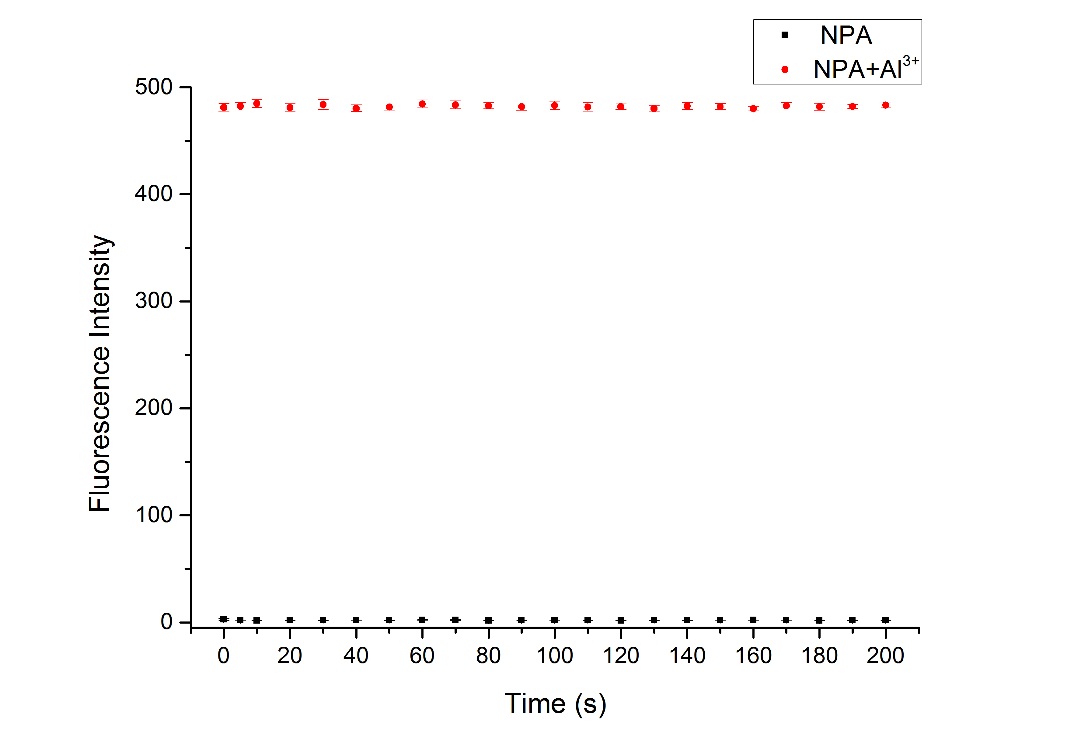


**Supplementary Figure 6.** Effect of time on the fluorescence intensity of **NPA** (10 μM) in the absence and presence of 5 equiv. Al^3+^(λ_ex_=400 nm). (Error bar was represented as mean ± standard deviation, n=3)


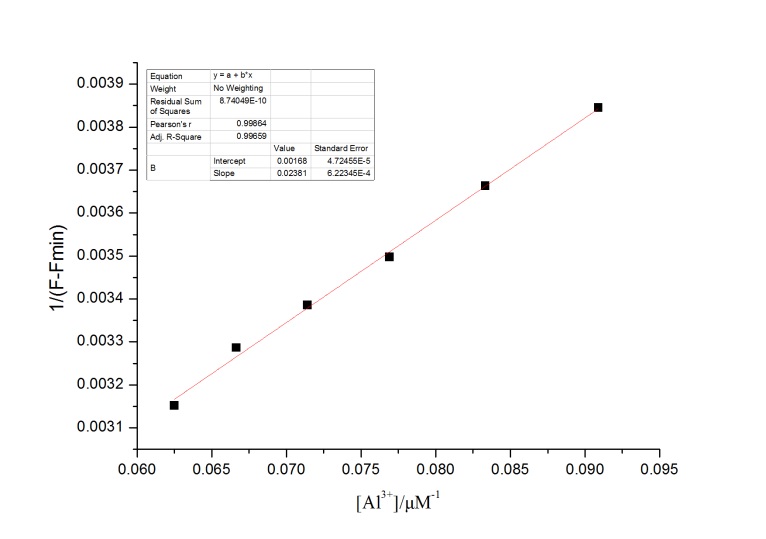


**Supplementary Figure 7.** Benesi-Hildebrand plot of **NPA** (10 μM) assuming a 1:1 stoichiometry for association between **NPA** and Al^3+^ in CH_3_OH by fluorescence spectroscopy.

^
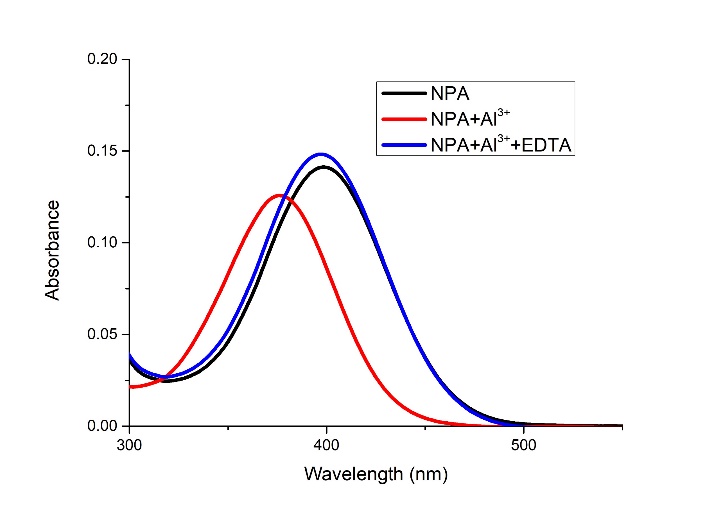
^

**Supplementary Figure 8.** UV-vis absorbance reversibility of **NPA** binding with Al^3+^ in CH_3_OH while adding 3 eq Al^3+^and 6 eq EDTA to the sensor solution.


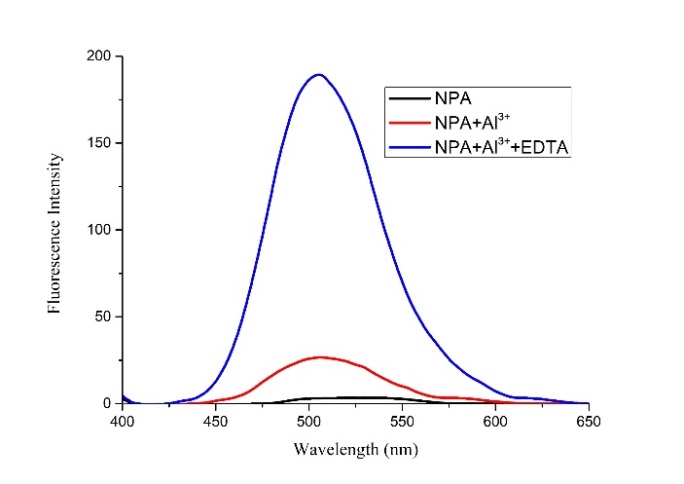


**Supplementary Figure 9.** Fluorescence intensity reversibility of **NPA** (10 μM) binding with Al^3+^ in CH_3_OH while adding 3 eq Al^3+^ and 6 eq EDTA to the sensor solution. λ_ex_=400 nm.

**
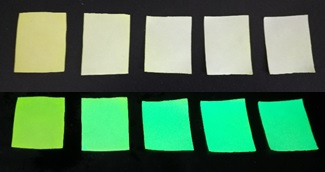
**

**Supplementary Figure 10.** Color change of test paper containing **NPA** (100 μM) in methanol solutions containing increasing amounts of Al^3+^ (0 μM, 100 μM, 200 μM, 300 μM, 400 μM, from left to right). Top: undera ZF-1A ultraviolet analyzer (Shanghai Qinke Instrument Equipment Co., Ltd.), bottom:under natural light


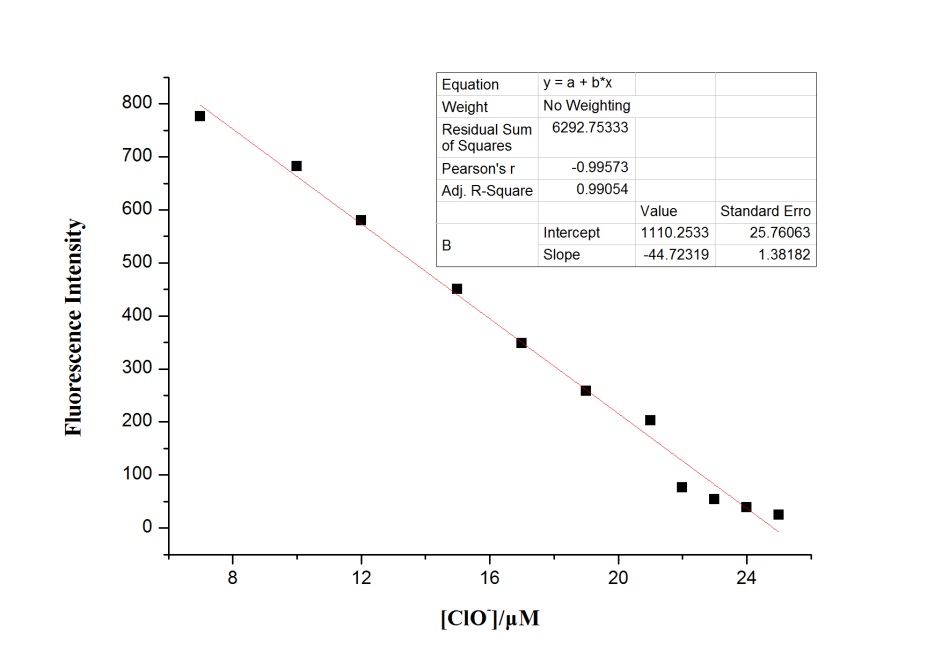


**Supplementary Figure 11.** The linear manner of fluorescence intensity of verse ClO^-^ concentration in CH_3_OH.


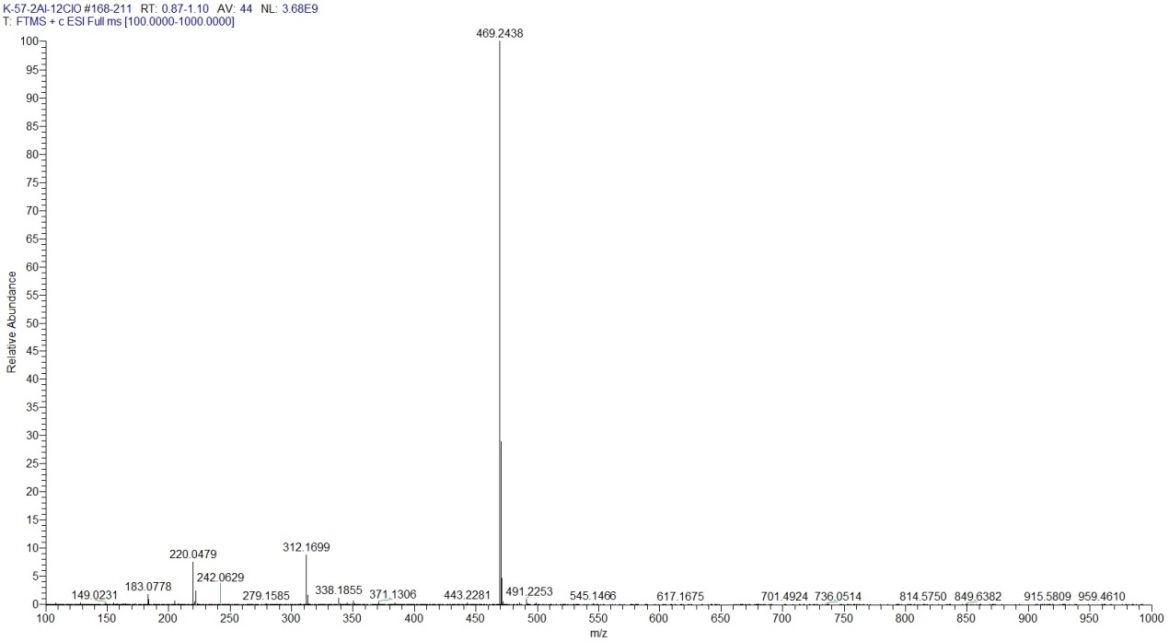


**Supplementary Figure 12.** ESI–MS spectrum of **NPA-Al^3+^** complex upon addition of ClO^-^ in CH_3_OH.


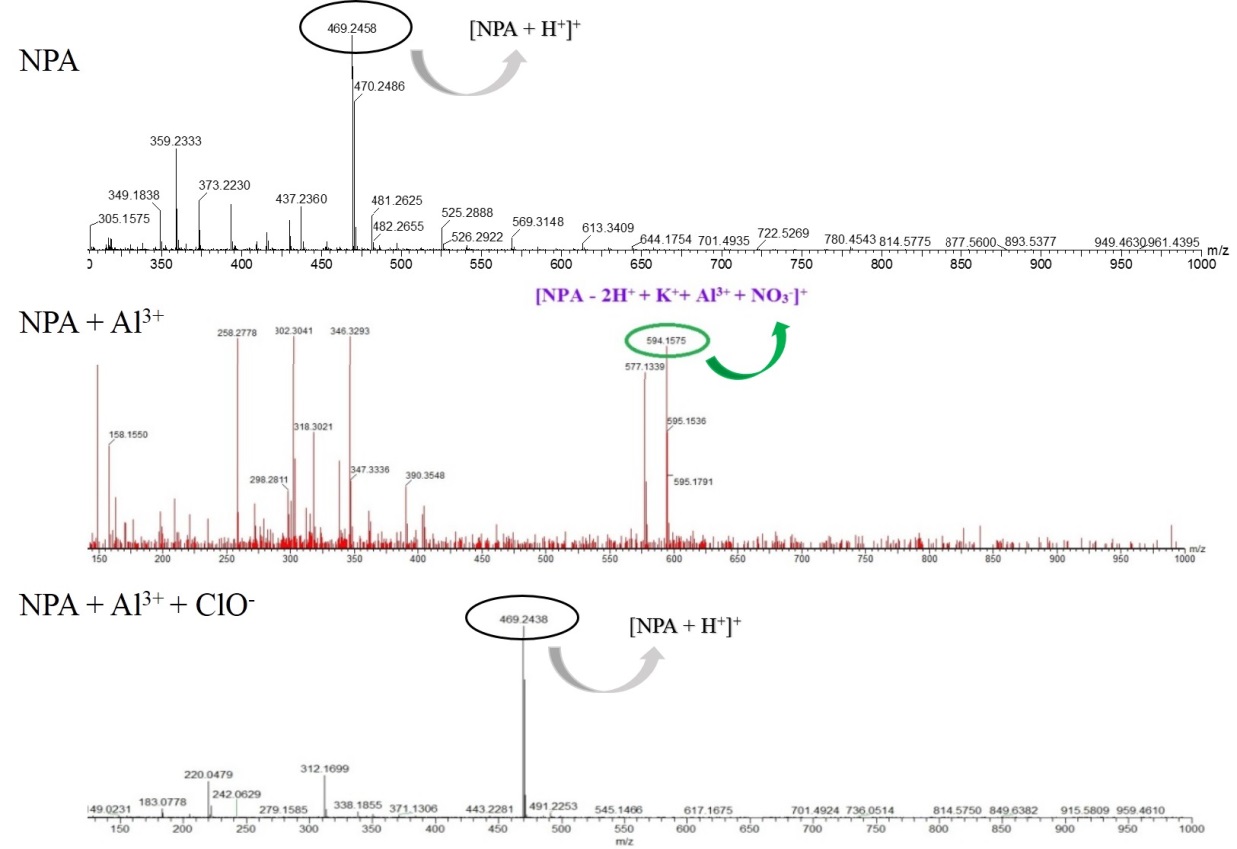


**Supplementary Figure 13.** ESI–MS spectrum of **NPA**, **NPA-**Al^3+^ and **NPA-**Al^3+^ complex upon addition of ClO^-^ in CH_3_OH, respecrively.


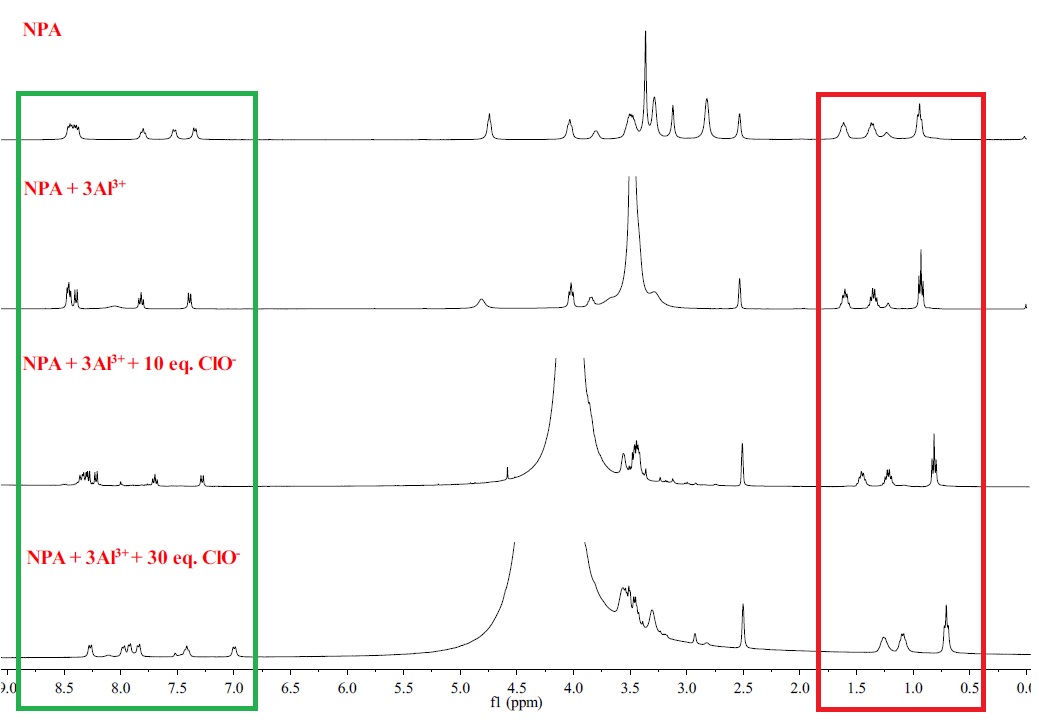


**Supplementary Figure 14.** Titration ^1^H NMR spectra of **NPA** and in the presence of Al^3+^ and ClO^-^ in DMSO *d*_6_.


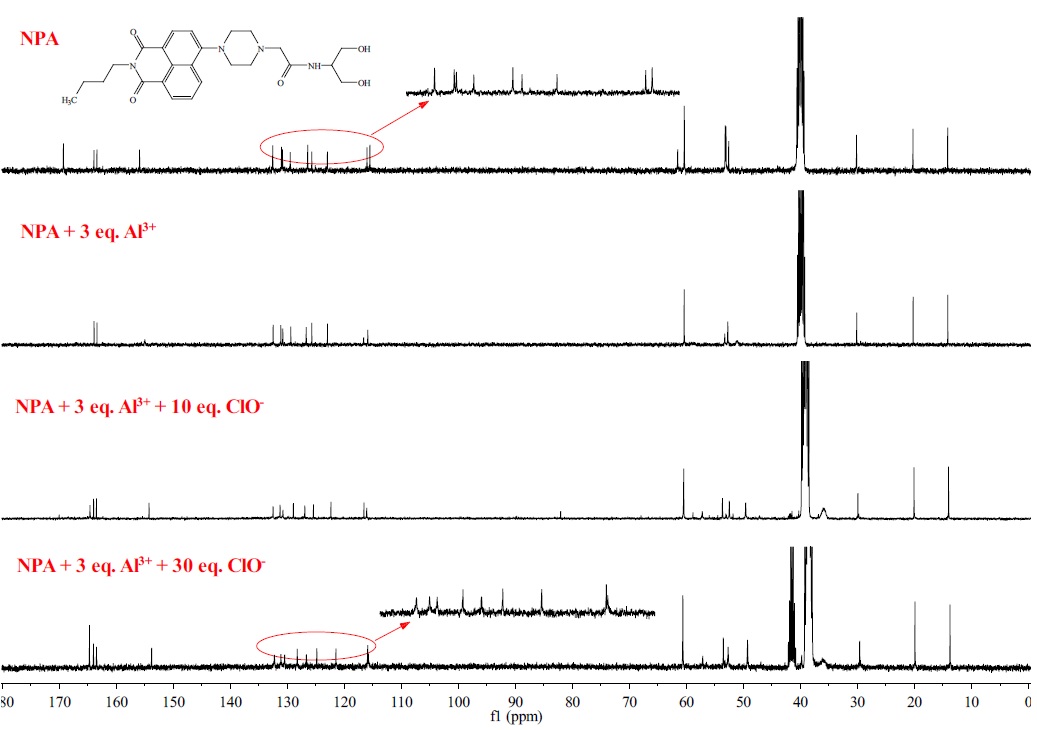


**Supplementary Figure 15.** Titration ^13^C NMR spectra of **NPA** and in the presence of Al^3+^ and ClO^-^ in DMSO *d*_6_.


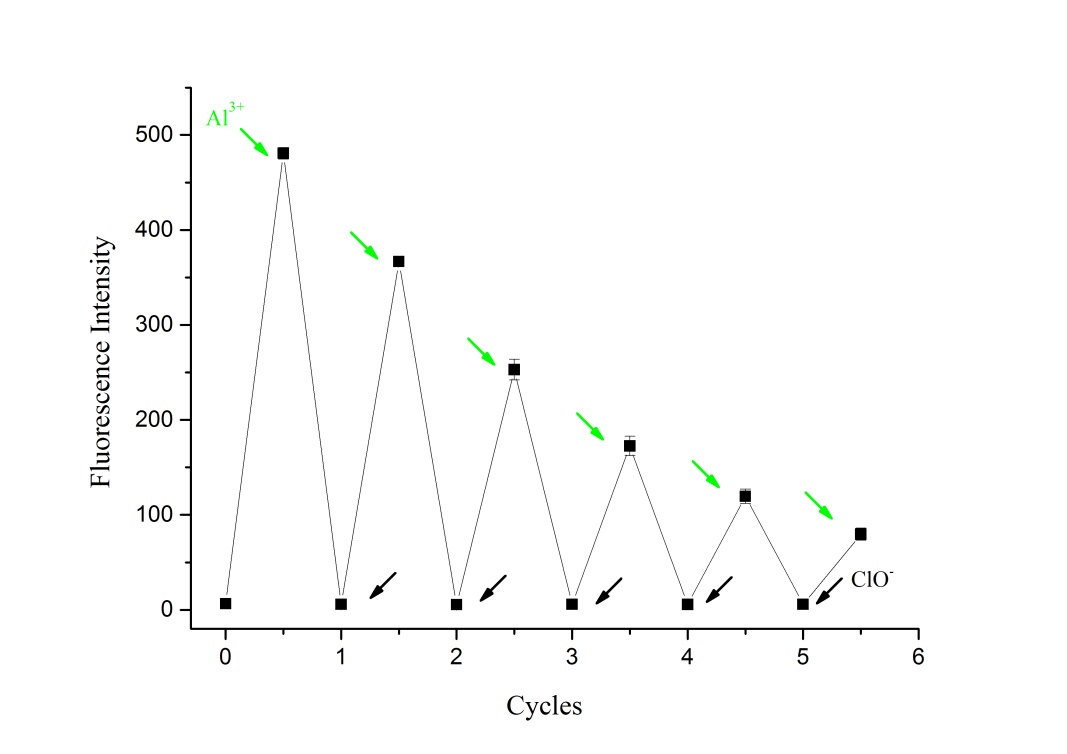


**Supplementary Figure 16.** Cycles of the fluorescence “off-on-off” response for Al^3+^ and ClO^-^ (Error bar was represented as mean ± standard deviation, n=3)

## Supplementary Tables

**Supplementary Table 1** Determination of Al^3+^ in water samples.

| Water samples  studied | Amount of standard  Al^3+^ added (μM) | Total Al^3+^ found (n=3) (μM) | Recovery of Al^3+^ (n=3) added (%) | RSD  (%) | Relative  error（%） |
| --- | --- | --- | --- | --- | --- |
| ultrapure water | 0.8 | 0.78 | 97.50 | 2.01 | -2.50 |
|  | 0.9 | 0.88 | 97.78 | 3.56 | -2.22 |
|  | 1.0 | 0.98 | 98.00 | 2.51 | -2.00 |
| Tap water (Department of  Chemistry) | 0.8 | 0.76 | 95.00 | 2.52 | -5.00 |
|  | 0.9 | 0.93 | 103.33 | 2.70 | 3.33 |
|  | 1.0 | 1.06 | 106.00 | 3.51 | 6.00 |

**Supplementary Table 2** Comparison of the characteristics of **NPA** with previously reported sensors.

| Ref | Detection method | Detection mode | Medium | Analyte | LOD | Binding  constant |
| --- | --- | --- | --- | --- | --- | --- |
| [32] | Fluorescence | OFF-ON-OFF | CH_3_CN | Al^3+^  Cr^3+^ | 5.47 ×10^-7^ M  2.76 × 10^-6^ M | NR  NR |
| [33] | Colorimetric | OFF-ON-OFF | CH_3_CN | Al^3+^  F^-^ | 2.16 × 10^-6^ M  NR | 3.45 × 10^3^ M^-1^  NR |
| [34] | Fluorescence | OFF-ON-OFF | H_2_O | Al^3+^  F^-^ | 1.35 × 10^-9^ M  NR | 5.0 × 10^6^ M^-1^  NR |
| [35] | Fluorescence | OFF-ON-OFF | CH_3_OH | Al^3+^  F^-^ | 9.24× 10^-7^ M  1.4 × 10^-5^ M | 9.1 × 10^4^ M^-1^  NR |
| [36] | Colorimetric | OFF-ON-OFF | Acetonitrile | F^-^/AcO^-^  Al^3+^ | 1.0025×10^−7^M/0.79×10^−7^ M  NR | NR  NR  NR |
| [37] | Fluorescence | OFF-ON-OFF | CH_3_CN:H_2_O  (7: 3 v/v) | Al^3+^  N_3_^-^ | 5.9×10^-8^ M  2.7×10^-7^ M | 6.42×10^4^ M^-1^  NR |
| [38] | Fluorescence | OFF-ON-OFF | CH_3_OH:H_2_O  (1: 1v/v) | Al^3+^  PO_4_^3-^ | 10^-5^ M  NR | 1.38×10^5^ M^-1^  NR |
| [39] | Fluorescence | OFF-ON-OFF | DMSO-H_2_O  (1:9, v/v) | Al^3+^  Cu^2+^ | 2.71×10^-5^ M  0.95×10^-6^ M | NR  NR |
| [40] | Fluorescence | OFF-ON-OFF | DMSO: H_2_O  (1:149, v/v, pH =7.0) | Al^3+^  PPi | 9.24×10^-6^ M  2.05×10^-5^ M | 2.32×10^-2^ M  3.70×10^3^ M^-1^ |
| [41] | Fluorescence | OFF-ON-OFF | CH_3_OH/H_2_O  (9:1, v/v, pH=7.0) | Al^3+^  CO_3_^2-^ | 4.30×10^-6^ M  5.60×10^-6^ M | NR  NR |
| [42] | Fluorescence | ON-OFF-ON | DMSO:H_2_O  (1:5, v/v, pH=7.2) | Al^3+^  F^-^ | 1.05 × 10^-8^ M  0.98 × 10^-8^ M | 8.5 × 10^5^ M^-1^  NR |
| [43] | Fluorescence | ON-OFF-ON | CH_3_OH:H_2_O  (1:500,v/v, pH=7.0） | Al^3+^  F^-^ | 9.2 × 10^-8^ M  1.12×10^-7^ M | NR  NR |
| [44] | Fluorescence | ON-OFF-ON | DMF/H_2_O  (1:9,v/v, pH=7.4) | Ni^2+^  Al^3+^ | 6.03×10^-8^ M  1.857×10^-6^ M | 7.14×10^6^ M^-1^  NR |
| This work | Fluorescence | OFF-ON-OFF | CH_3_OH | Al^3+^  ClO^-^ | 2.03×10^-8^ M  4.43×10^-8^ M | 7.06×10^4^ M^-1^  NR |

LOD: The limit of detection; NR: Not reported in the corresponding paper.

**Supplementary Table 3** Comparison of the **NPA** with previously reported sensors with lower binding constant than **NPA**.

| Ref | Detection method | Analyte | LOD | Binding constant |
| --- | --- | --- | --- | --- |
| [1] | Fluorescent | Al^3+^  N_3_^-^ | 5.90 × 10^-8^ M  2.70 × 10^-7^ M | 6.42 × 10^4^ M^-1^ |
| [2] | Fluorescent | Al^3+^  Zn^2+^ | 3.70 × 10^-9^ M  3.00 × 10^-8^ M | 1.16 × 10^4^ M^-1^  2.08 × 10^4^ M^-1^ |
| [3] | Fluorescent | Al^3+^  F^-^ | 1.10 × 10^-7^ M  1.47 × 10^-6^ M | 7.76 × 10^3^ M^-1^ |
| **This work** | Fluorescent | Al^3+^  ClO^-^ | 2.03 × 10^-8^ M  2.34 × 10^-8^ M | 7.06 × 10^4^ M^-1^ |

**References**

[1] Rai, A., Kumari, N., Srivastava, A. K., Singh, S. K., Srikrishna, S., and Mishra, L. (2016). Rhodamine hydrazone as OFF–ON–OFF type selective sequential sensor of Al^3+^ and N^3-^ ions. ***J. Photochem. Photobiol. A Chem****.* 319, 78–86. doi: 10.1016/j.jphotochem.2016.01.003

[2] Li, Y., Niu, Q., Wei, T., Li, T. D. (2019). Novel thiophene-based colorimetric and fluorescent turn-on sensor for highly sensitive and selective simultaneous detection of Al^3+^ and Zn^2+^ in water and food samples and its application in bioimaging. ***Anal. Chim. Acta*** 1049, 196-212. doi: 10.1016/j.aca.2018.10.043

[3] Wu, Y. P., Rahman, F. U., Bhatti, M. Z., Yu, S. B., Wang, H., Li, Z. T., et al. (2013) Acylhydrazone as a Novel "Off-On-Off" Fluorescence Probe for sequential detection of Al^3+^ and F^‒^. ***New J. Chem****.* 42, 14978-14985. doi: 10.1039/x0xx00000x
